# Review of the biomechanics and biotribology of osteochondral grafts used for surgical interventions in the knee

**DOI:** 10.1177/0954411915615470

**Published:** 2015-12

**Authors:** Philippa Bowland, E Ingham, Louise Jennings, John Fisher

**Affiliations:** Institute of Medical and Biological Engineering, University of Leeds, Leeds, UK

**Keywords:** Osteochondral graft, biotribology, biomechanics, knee, articular cartilage, subchondral bone, friction, scaffold, tissue engineering, regeneration

## Abstract

A review of research undertaken to evaluate the biomechanical stability and biotribological behaviour of osteochondral grafts in the knee joint and a brief discussion of areas requiring further improvement in future studies are presented. The review takes into consideration osteochondral autografts, allografts, tissue engineered constructs and synthetic and biological scaffolds.

## Introduction

Osteoarthritis is a prevalent degenerative joint disease of the synovial joints affecting 8.75 million people in the United Kingdom alone. Patients suffering with osteoarthritis of the knee account for just over half (4.71 million) of all individuals living with osteoarthritis in the United Kingdom. The prevalence of knee osteoarthritis and the associated socioeconomic pressures it presents are set to increase in the future; accounting for predicted increases in population obesity, growth and ageing, the incidence of osteoarthritis of the knee in the UK population is estimated to have nearly doubled by 2035 (source: www.arthritisresearchuk.org.uk).

Osteochondral defects on the articulating surfaces of the knee typically occur due to traumatic injuries, abnormalities in the subchondral bone (osteochondritis dissecans and avascular necrosis) and chronic mechanical overload due to factors such as severe joint misalignments and the removal of meniscal tissue.^1^ Osteochondral defects disrupt the local biomechanics and biotribology of the joint, and if left untreated will persist indefinitely, resulting in further degenerative wear of the articulating surfaces leading to the onset of osteoarthritis. Total knee replacement is the most common type of surgery used to treat established cases of osteoarthritis; almost 80,000 primary knee joint replacements were implanted in 2012 in the United Kingdom.^2^ Despite being regarded as a highly successful and cost-effective treatment, total knee joint replacements have a finite longevity and may require multiple revisions during the patient’s lifetime.^2,3^ Moreover, while total knee replacements are effective in relieving pain, full function and range of activities are not always restored.

A wide variety of surgical methods for the treatment of osteochondral defects are currently available (Table 1); these range from purely palliative treatments such as arthroscopic debridement to treatments which aim to stimulate fibrous repair tissue (e.g. microfracture), those utilising whole tissue transplantation (e.g. osteochondral autografts and allografts) and finally cell-based approaches (e.g. autologous chondrocyte implantation (ACI)).

**Table 1.** Overview of current surgical methods for the treatment of osteochondral defects in the knee.

Surgical treatment	Advantages	Limitations
Arthroscopic debridement and lavage	Arthroscopic or minimally invasive	Progressive deterioration Recurring symptoms
	Cost-effective	
	Short rehabilitation time	
Microfracture or marrow stimulation	Cost-effective Surgically reproducible	Fibrocartilage formation
		Partial defect filling
		Functional deterioration after 18–24 months^4^
Osteochondral autograft transplantation and mosaicplasty	Restoration of hyaline cartilage articulating surface Good chondrocyte survival rateGood clinical results at medium long-term follow-up^5^	Lack of cartilage integration
		Poor matching of graft and host cartilage congruency
		Donor site morbidity
		Limited tissue availability
		Potential chondrocyte apoptosis during graft impaction^6,7^
Osteochondral allograft transplantation	Restoration of hyaline cartilage articulating surface	Potential immunological response and disease transmission
	Treatment of large defects	Limited graft availability
	Good long-term clinical results and graft survival^8^	Potential chondrocyte apoptosis during graft impaction^6,7^
Autologous chondrocyte implantation (ACI) and matrix-assisted ACI (MACI)	Arthroscopic or minimally invasivePotential for hyaline cartilage repair tissue Use of autologous cells	Expensive
		Two-stage procedure
		Variable repair tissue type: hyaline like, fibrocartilage and mixed^9^
		Limited defect filling and integration^10^

The clinical application of osteochondral grafts in the knee currently involves the implantation of single or multiple (mosaicplasty) autologous or allogeneic grafts. The aim of osteochondral graft implantation is to achieve a congruent articular surface resembling that of the native joint in order to restore the biomechanics and biotribology of the joint. The current clinical use of osteochondral autografts and allografts is limited by a number of factors, including (1) tissue availability, (2) donor site morbidity, (3) disparity in congruency between graft and host tissues and (4) lack of integration between graft and host articular cartilage.^11,12^ Cell-based approaches to the treatment of osteochondral defects, such as ACI and matrix-assisted ACI utilising scaffolds have also demonstrated a number of inherent limitations on clinical follow-up. These limitations include (1) fibrocartilage formation, (2) incomplete defect filling and (3) limited integration with surrounding tissues.^10,13^

Tissue engineering of osteochondral constructs has the potential to overcome the limitations of existing therapies and provide surgical solutions with improved long-term outcomes. The design of tissue engineered constructs is often based on a combination of three fundamental elements, namely, scaffolds, cells and bioactive molecules, with the aim of producing functional tissues in vitro or in vivo.^14^ By engineering an osteochondral construct, constructs may be developed with biological, structural, biomechanical and tribological properties that closely mimic those of natural cartilage and bone, which are essential for long-term performance and durability in the natural knee joint. To date, no tissue engineered osteochondral construct has yet regenerated functional tissues that possess the properties of native cartilage and bone.

A number of approaches have been adopted in the research and development of a potential regenerative solution for osteochondral replacement; these include synthetic and natural scaffolds pre-seeded with cells in vitro or as intelligent scaffolds capable of in vivo regeneration utilising the body’s own endogenous cells. Scaffolds may be monophasic, biphasic, triphasic or multiphasic in structure, consisting of one or more layers or scaffold materials (Table 2) with differing material properties and architecture. Varying cell types and growth factors may be introduced into each layer to encourage the regeneration of cartilage and bone tissue.

**Table 2.** Overview of materials commonly used in the development of regenerative osteochondral scaffolds.^14–17^

Scaffold classification	Material
Natural polymers	Collagen
	Gelatin
	Fibrin
	Hyaluronic acid
	Alginate
	Agarose
	Chitosan
	Silk
Synthetic polymers	Poly(ethylene glycol) (PEG)
	Poly(caprolactone) (PCL)
	Poly(lactic acid) (PLA)
	Poly(glycolic acid) (PGA)
	Poly(lactic-*co*-glycolic) acid (PLGA)
Bioceramics	Bioactive glasses
	Hydroxyapatite
	Calcium phosphates
Extracellular matrix	Decellularised and devitalised cartilage and bone tissue
Combination of scaffolds	Combination of materials as stated above

The predicted future population trends regarding ageing, obesity and osteoarthritis and the limitations in current therapies for the treatment of osteochondral defects indicate a clear requirement for the development of effective early stage interventions to repair or regenerate osteochondral defects in the knee. Regenerative solutions for osteochondral defect repair have the potential to delay or halt further degenerative changes and may ultimately negate the requirement for total joint replacements in the long term. This review aims to present the research undertaken to assess and evaluate the biomechanics and biotribology of osteochondral autografts, allografts, tissue engineered constructs and scaffolds for the repair or regeneration of osteochondral defects in the knee.

Two major challenges exist for the successful application of osteochondral grafts and novel regenerative solutions, the first being the restoration of biomechanical and biotribological function in order to establish the correct environment for tissue repair and regeneration, which is the primary focus of this review. The second challenge is the stratification of the population and the development of segmented product interventions designed with appropriate levels of precision, which can be delivered to reliably restore function and performance. This is addressed as a future challenge in the discussion.

## Biomechanics and stability of osteochondral grafts

The aim of osteochondral grafts is to restore the congruent articulating surfaces of the joint, restoring normal joint biomechanics and biotribology (the biphasic load carriage and lubrication). Achieving and maintaining the congruent articular surfaces, along with the integrated support from the underlying bone are paramount to the long-term success of osteochondral graft procedures and the prevention of further progressive degenerative changes in the joint. Graft stability in the initial period following implantation is dependent on the resistance to motion arising from the graft–host interference fit and where present, support from the underlying trabecular bone structure. The graft–host interference fit (press-fit) is a direct product of the material properties and geometries of the graft and the host implantation site. Grafts that protrude above or subside below congruency level following implantation may induce inferior biomechanical and tribological conditions in the joint, potentially resulting in the onset of degenerative changes.

**Figure 1.**
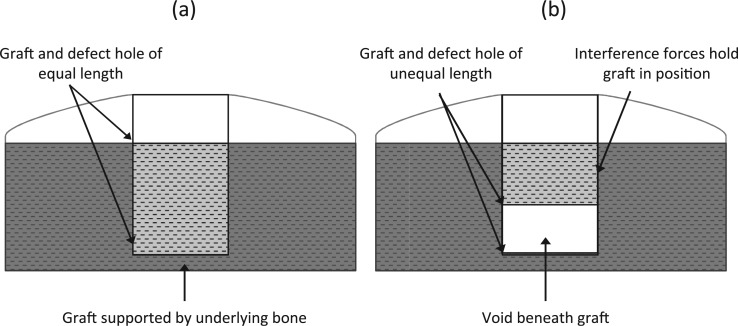
Schematic diagram of osteochondral graft and defect hole geometries: (a) bottomed graft (osteochondral graft and defect hole of equal length) and (b) unbottomed graft (osteochondral graft shorter in length than defect hole).

Biomechanical studies that have evaluated the effects of graft and defect geometry have shown that the primary stability of osteochondral autografts or allografts in the initial post implantation period is greater when the graft and defect length are equal (bottomed grafts; see Figure 1).^18,19^ These studies have evaluated graft stability by measuring the compressive push in forces required to displace grafts a set depth below congruency level with the surrounding host cartilage.

The stability of bottomed grafts (Figure 1(a)) is greater than that of unbottomed grafts (Figure 1(b)) due to support from the underlying subchondral bone; unbottomed grafts rely solely on the graft–host interference forces present to secure them in position in the post-operative period. The stability of unbottomed grafts increases with increasing surface area in contact with the host; therefore, a larger graft surface area provides greater resistance to motion due to the greater graft–host interference forces present. Graft surface area may be increased for unbottomed grafts by increasing graft diameter for a fixed length or conversely by increasing graft length for a fixed diameter. The results of Kock et al.^18^ and Kordás et al.^19^ showed that when grafts are inserted into defects of greater length (unbottomed grafts), grafts with larger diameters resist greater push in forces; similarly, graft stability also increases with increasing graft length with unbottomed grafts. Conversely, shorter bottomed grafts have been shown to provide greater resistance to push in forces than longer bottomed grafts.^18^

Investigations that have assessed graft pull out strength have measured the resistance to graft movement due to the graft–host interference forces. The study conducted by Duchow et al.^20^ concluded that shorter grafts and those of smaller diameter resisted significantly lower pull out loads; these results are in agreement with those obtained by Kock et al.^18^ and Kordás et al.^19^ for unbottomed grafts during push in tests. In vivo tests in both human and animal models have also demonstrated that the lack of basal support in unbottomed grafts is likely to predispose them to a tendency to subside below congruency level.^21,22^

Finite element simulations conducted by Wu et al.^23^ showed that the implantation of congruent osteochondral grafts into the femoral condyle resulted in altered stress and strain distributions in the opposing cartilage surface when compared to an intact joint. A discontinuous contact stress profile over the graft–host interface was present when the grafts were inserted congruent to the native cartilage surface. The differences in the contact stress profiles are attributable to the discontinuous cartilage surface and may negatively affect the development of repair tissue in the graft–host boundary space. The finite element simulations also highlighted that there was an abnormal local tensile stress present in the opposing articulating surface of the tibial plateau when grafts were inserted either proud or countersunk to the host cartilage layer; such abnormal stresses may compromise the integrity of the opposing cartilage surface resulting in degenerative changes.

The effects of osteochondral defects on the contact stress of the surrounding articular cartilage have been studied experimentally. Elevated contact stresses have been shown to occur in the rim of osteochondral defects with peak stresses and increased contact stress gradients also occurring in the cartilage surrounding the defect.^22,24,25^ Kock et al. showed that the implantation of osteochondral grafts in a mosaicplasty procedure reduced the contact stress present around the edge of osteochondral defects. Furthermore, the study indicated that similar to untreated osteochondral defects, unbottomed grafts that had subsided below congruency also had increased rim stresses compared to implanted grafts that had remained congruent.^22^ Gratz et al.^26^ reported increased axial, lateral and shear strains in cartilage adjacent to defects and slightly elevated shear strains in the opposing cartilage surfaces. Grafts experiencing subsidence, similar to untreated osteochondral defects, are likely to result in altered stress–strain distributions in the surrounding and opposing articular cartilage surfaces. Elevated stress–strain levels and abnormal distributions may place the articulating cartilage surfaces of the knee joint at risk of damage through biomechanical and mechanobiological mechanisms; therefore, it is important that new graft designs have adequate material properties to provide suitable resistance to motion and loading.

**Figure 2.**
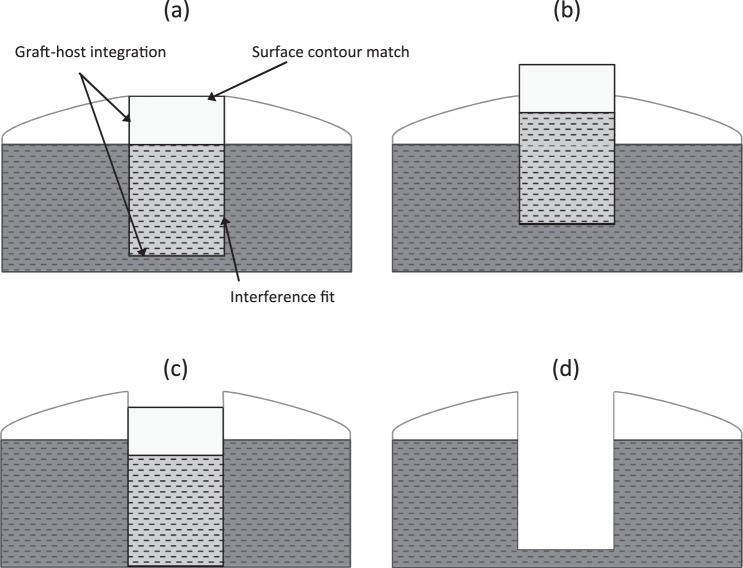
Schematic diagram of possible outcomes following osteochondral graft implantation: (a) ideal implantation scenario for restoring congruent articular surface, (b) osteochondral graft implanted proud of host articular cartilage surface, (c) osteochondral recessed below host cartilage surface and (d) no osteochondral graft implanted into defect.

Investigations by Koh et al.^27,28^ have demonstrated significant increases in contact pressure when grafts are inserted proud (Figure 2(a)) of the cartilage surface or are inserted in an angled fashion. Osteochondral grafts implanted 1 and 0.5 mm proud of the femoral cartilage surfaces were shown to significantly increase contact pressure compared to grafts inserted to flush level by 57% and 48%, respectively. Similarly, grafts recessed (Figure 2(b)) 0.5 and 1 mm below flush level were also subject to significantly increased contact pressures when compared to intact cartilage.

Nakagawa et al.^29^ conducted a post-surgical arthroscopic evaluation of individuals with protruding or recessed osteochondral autografts at the time of mosaicplasty surgery (mean follow-up period 14.8 months). Follow-up arthroscopies showed that protruding plugs displayed fibrillation at the graft edges and degenerative changes in the opposing tibial surfaces including cartilage softening and fibrillation. Studies evaluating the effects of altered joint biomechanics due to protruding osteochondral grafts indicate a clear relationship between altered contact mechanics and subsequent damage of the articulating cartilage surfaces; this is likely due to resultant changes in the local biotribology of the joint.

Several animal studies examining the outcomes of single osteochondral graft transfer and mosaicplasty procedures have indicated integration of the subchondral and underlying trabecular bone with the surrounding host bone at 3–6 months postoperatively.^30–33^ Fibrocartilage was also shown to have grown above the subchondral bone level in the void between the periphery of the graft and the host tissue. However, fibrocartilage in-growth was inconsistent with some specimens displaying clefts in the repair tissue from the cartilage surface down to the subchondral bone level at the graft–host cartilage interface.^30–34^

Unbottomed grafts, due to a lack of support from underlying bone, rely predominantly on the interference fit to resist subsidence below congruency in the post-operative period until integration with underlying bone occurs. A number of studies performed in ovine models to investigate the effects of osteochondral graft alignment and subsidence have shown that when grafts subside to expose the subchondral host bone of the defect walls, the articular cartilage surface of the graft is susceptible to fibrous tissue overgrowth.^21,33,34^ The studies by Huang et al.^34^ and Nosewicz et al.^21^ indicated that when grafts subside less than 1 mm below congruency, cartilage thickening may occur, compensating for the difference between the surface profiles of the graft and host cartilage. Subsidence of grafts below 1 mm has been shown to induce significant fibrocartilaginous overgrowth; despite this, the surface profiles of the graft and host often still remain incongruent.^21,33,34^ These results correlate with the clinical observations of Nakagawa et al.,^29^ where all grafts that had subsided greater than 1 mm below congruency displayed fibrocartilage overgrowth. The presence of fibrocartilage on the articulating joint surfaces is undesirable as fibrocartilage is known to be biomechanically and histologically inferior to articular cartilage; this may result in the onset of degenerative changes in the cartilage of the graft and surrounding host tissue.^35–37^

The evaluation of the biomechanics of osteochondral autografts, allografts and tissue engineered constructs is limited in the published literature. Studies designed to assess the in vivo and in vitro development of tissue engineered osteochondral constructs have focused predominantly on morphological and histological scoring and assessment as opposed to mechanical and tribological functionality. Jeon et al.^38^ reviewed studies between 2009 and 2012 that were concerned with the evaluation of osteochondral repair by constructs implanted into animal models. This review indicated that only 15% of the studies reviewed reported mechanical evaluation of explants following in vivo implantation. Lopa and Madry^39^ in their review of preclinical studies applying biphasic osteochondral scaffolds also reported limited numbers of studies reporting biomechanical analysis of explants to assess osteochondral repair. Where mechanical testing is reported, this tends to be limited to basic indentation, stiffness and stress relaxation tests of the explanted grafts without consideration of the effects of the grafts on the whole joint system.

## Biotribology (including biphasic mechanics and lubrication) of osteochondral grafts

Osteochondral grafts consist of an articular cartilage component and an underlying supporting bone component that also serves to anchor and constrain the articulating hyaline cartilage interface of the graft and establish the essential thin layer constrained contact mechanics in the articular cartilage layer. Cartilage is a biphasic tissue with a complex zonal structure and composition; the structure and composition of cartilage endow the tissue with exceptional functional properties allowing low friction movement under high load bearing conditions. The complex organisation of collagen type II fibres and hydrophilic proteoglycans in a dense cross-linked network results in the retention of interstitial fluid within the tissue, under the severe loading conditions in the natural knee. Load is initially carried by the fluid phase in cartilage tissue, facilitated through an increase in internal fluid pressure; this results in a very low coefficient of friction.^40^ The cartilage layer, integrated to the underlying bone, is primarily responsible for the biotribological function of osteochondral grafts in the natural joint environment and the subsequent maintenance or disruption of the biotribology in the surrounding and opposing cartilage surfaces.

The main aim of osteochondral grafts is to reconstruct the natural articulating surface and biphasic biotribology of the joint and restore low friction articulation, in order to resist degeneration and wear. For osteochondral grafts to be successful, they must possess adequate tribological and mechanical properties to withstand the complex loading environment within the joint. The structure, composition and subsequent material properties of osteochondral grafts (the integrated structure to replace the bone and the cartilage) must first be sufficient in the short term for the graft to support the growth and integration of repair tissue under complex loading in the joint. Second, the biomechanical and biotribological properties of the graft and repair tissues should not compromise the integrity of the surrounding and opposing cartilage surfaces. Osteochondral grafts aim to repair the underlying supporting bone structure and restore a near frictionless articulating surface; therefore, the biotribological performance of these grafts in the natural joint is a key factor in determining their success.

Biotribological evaluation is used to simultaneously study the friction, lubrication and wear of materials under compressive loading and sliding shear stress; the biotribological properties of a graft may provide a better indication of functionality compared to the uniaxial biomechanical properties alone. Pin-on-plate tribological test methods (while not replicating the geometry or complex motions of the natural knee) have commonly been used to study the tribology of cartilage.^41–45^ The use of these test methods has also been extended to study the tribology of cartilage scaffold biomaterials and tissue engineered cartilage constructs.^40,46–51^ Pin-on-plate test methods involve the translation and/or rotation of a pin against a larger counterface in the presence of a lubricant. Commonly used counterface materials are natural cartilage–bone plate specimens or materials such as stainless steel and glass. Small-scale in vitro pin-on-plate test methods allow for the control of experimental variables such as normal load, sliding distance and velocity, contact pressure and tissue loading and unloading intervals which dictate the outcomes under investigation such as friction and wear.^52^ These simple small-scale in vitro tests can be utilised to assess the tribological performance of newly developed biomaterials and engineered tissues at an early stage. The knowledge gained from pin-on-plate test methods may also be used to better understand the tribological behaviour of complex whole joint simulation models that are capable of reproducing the natural physiological conditions experienced in synovial joints such as the knee.^52^

Analysis of osteochondral graft insertion into the knee joint using finite element simulations has indicated that the implantation of grafts results in altered stress and strain distributions in the cartilage surfaces, with discontinuous stress profiles noted in the region of the graft and host tissue interface.^23^ When implanting osteochondral grafts, it is particularly difficult to achieve a perfectly congruent articulating surface; therefore, it is likely that even with grafts inserted to flush level, there may be subsequent increased wear in the joint due to an increased coefficient of friction arising from a discontinuous articulating surface (edge effects).

Lane et al.^53^ studied the effects of osteochondral allograft implantation on the coefficient of friction of cartilage in a caprine knee model in vitro. The study highlighted a significant increase in coefficient of friction when no graft was inserted into the defect and when grafts were inserted flush, protruding and recessed in respect to the host cartilage surface (Figure 2). The greatest increase in coefficient of friction was observed in grafts that were protruding; the measured coefficient of friction in this group was four times (0.075 ± 0.040) that of normal cartilage (0.016 ± 0.006). The empty defect (0.033 ± 0.023) and the recessed grafts (0.036 ± 0.019) had a significantly lower coefficient of friction compared to the proud plug; however, no significant difference was observed between the proud and flush grafts (0.054 ± 0.041). As mentioned previously, protruding grafts in the knee joint have been observed to undergo fibrillation and induce degenerative changes in opposing cartilage surfaces.^29^

Bobrowitsch et al.^54^ analysed the differences between intact cartilage surfaces and those implanted with osteochondral allografts at differing heights with regard to the frictional response of cartilage and the resulting contact pressure. The study utilised an ovine carpometacarpal joint model in vitro, and the frictional characteristics of the joint were assessed using the dissipated energy method as described by Walter et al.^55^ The results, similar to those observed by Lane et al.,^53^ indicated higher levels of friction when the defect was left empty and when grafts were implanted flush and recessed to the surrounding cartilage. In contrast to previous studies, the high implanted grafts did not show a significant increase in friction; however, the friction was seen to increase dramatically when the cartilage surface of the high implanted grafts was damaged. The joint contact area was shown to predominantly decrease and the mean contact pressure increase in all treatment groups (empty defect, flush, recessed and protruding) compared to the intact joint. Following osteochondral graft implantation, cartilage damage was only observed on the edge of the graft and defect hole. A lack of integration between the graft and surrounding host cartilage on the articulating surfaces may result in increased friction and wear due to the effects of the tibia moving across the edge between the graft and host tissue. This may induce increased levels of friction and wear at the graft edges, on the host tissue adjacent to the graft and on the opposing articulating surfaces.

A limited number of studies have investigated the biotribology of tissue engineered cartilage and scaffold materials designed for the regeneration of articular cartilage.^40,46–50,56,57^ Previous in vitro experiments evaluating the friction and wear response of tissue engineered cartilage in pin-on-plate experimental set-ups have reported a higher equilibrium coefficient of friction compared to native cartilage.^47–49,57^ Several of these studies have shown a time-dependent frictional response of the tissue engineered cartilage constructs^40,46,47,49^ similar to that of native cartilage. The presence of a time-dependent frictional response is indicative of the presence of some biphasic behaviour and interstitial fluid pressurisation within the tissue engineered cartilage constructs. To date, however, tissue engineered cartilage constructs have yet to demonstrate the tribological function of natural cartilage.

Studies have shown that that the composition and structure of engineered tissues and scaffolds for osteochondral repair play a key role in dictating their frictional response and susceptibility to damage during biotribological testing.^40,46,47,49,56^ Whitney et al.^46^ compared the frictional response of scaffold-free tissue engineered cartilage constructs to bovine cartilage specimens in a pin-on-plate test configuration. The tissue engineered cartilage exhibited a time-dependent frictional response similar to that of native cartilage. The measured frictional force was initially low and then increased over time before appearing to approach equilibrium; however, at later time points, the frictional coefficient of friction of the tissue engineered specimen was seen to decrease. The average reported mean frictional shear stress was not significantly different between the two groups; despite this, all of the tissue engineered cartilage samples clearly showed evidence of surface peeling with 90% of samples delaminating before equilibrium was reached. Delamination of tissue engineered cartilage in the superficial zone has also been reported previously by Plainfosse et al.^40^ The tissue engineered cartilage constructs studied by Whitney et al.^46^ were found to have a significantly lower glycosaminoglycan and collagen content compared to native tissues; similarly, the shear and aggregate modulus of the tissue engineered specimens were approximately 60% that of their natural counterparts. Lima et al.^49^ also reported similarly low levels of collagen in the tissue engineered cartilage samples, therefore limiting the constructs ability for internal fluid pressurisation and the maintenance of a low coefficient of friction. Plainfosse et al.^40^ reported that levels of matrix components, particularly collagen type II, are commonly lower in tissue engineered cartilage compared to mature natural articular cartilage and may be considered structurally immature; the tissue immaturity is reflected in the low aggregate modulus often obtained during compression testing of such constructs. Investigations by both Plainfosse et al.^40^ and Whitney et al.^46^ reported an increasing coefficient of friction with time during testing followed by a notable decrease at later time points. The decrease in coefficient of friction may have arisen from the generation of wear debris and the potential accumulation of wear particles on the counterface plates, acting to reduce the surface roughness and therefore resistance to motion.^40,58^

Morita et al.^47^ also reported a time-dependent frictional response for cartilage that had been engineered utilising a fibrin scaffold. The equilibrium coefficient of friction reached was higher when compared with native cartilage; however, this was reported to decrease when the engineered cartilage was cultured for longer time periods. Increased culture time was associated with an increase in deposition of surface layer extracellular matrix and an increase in proteoglycan content allowing for improved retention of proteoglycans and interstitial fluid.

Acellular and cellular cartilage scaffolds have been shown to display differing levels of resistance to friction and wear during shear testing. Accardi et al.^56^ showed that the level of friction of acellular poly(ε-caprolactone) scaffolds under shear was predominantly dependent on surface morphology and fibre orientation, which in turn determined the onset and degree of damage sustained. For acellular scaffolds with aligned fibre orientations, it was shown that alignment of the scaffold fibres in the direction of shear, as is present in native cartilage collagen fibres, was preferential in order to increase the resistance to tension and damage due to shear. The fibre orientation in the cellular scaffolds did not appear to have a significant effect on their friction and wear characteristics due to a masking effect by the deposited extracellular matrix. These tissue engineered scaffolds did not exhibit the time-dependent frictional response typically seen in native cartilage; furthermore, the cellular scaffolds also demonstrated a higher equilibrium coefficient of friction compared to the acellular scaffolds. These factors were attributed to limited culture time, a lack of extracellular matrix deposition and a subsequently limited fluid load support. The reduced load bearing capacity of the tissue was thought to result in the formation of surface damage and wear debris leading to an increase in the surface roughness and coefficient of friction.

Studies investigating the frictional response of tissue engineered cartilage replacements^40,46,47,56^ indicate that in order to improve the frictional properties, it would be beneficial to more closely replicate the tissue structure and composition of extracellular matrix components such as collagen and proteoglycans; this may subsequently allow for improved biphasic behaviour and a low coefficient of friction.^40^ The complex fibre structure and orientation in natural cartilage with an orientated surface layer of fibres, which carries tensile stresses, have been shown to be necessary to sustain the hydrostatic stress field and fluid pressurisation in cartilage.

Biotribological assessment of osteochondral repair solutions in the literature is predominantly limited to small-scale pin-on-plate methodologies and basic whole joint torsion models. In order to progress the development of novel osteochondral repair solutions, such as tissue engineered constructs and their successful delivery to the clinic, effective preclinical evaluation is required in order to assess their efficacy in the short and long terms. The development of in vitro whole joint simulation models capable of reproducing the physiological and anatomical conditions of the natural joint may prove to be an invaluable preclinical testing tool.^52,54,59,60^ These whole joint simulation models can be utilised to study the friction and wear properties of potential osteochondral repair solutions under a variety of dynamic loading profiles simulating those experienced in the natural joint in vivo; furthermore, such models will allow for the effects of the osteochondral repair solution on the whole joint system to be evaluated. As highlighted by Jeon et al.,^38^ appropriate mechanical and tribological assessment of explants from in vivo animal test models in addition to purely histological methods should also be carried out in order to provide valuable information for the development of successful osteochondral repair solutions.

## Summary

The restoration of a low friction congruent articulating surface and the stability of osteochondral repair solutions are key factors in avoiding the introduction of abnormal stress and strain distributions in the surrounding and opposing cartilage surfaces. Research has indicated a clear link between protruding grafts and cartilage wear and degeneration; similarly, recessed grafts have been shown to induce similar stress and strain changes in the adjacent and opposing articulating surfaces as untreated osteochondral defects. The subsidence of osteochondral grafts below flush level following implantation has also been shown to induce significant fibrocartilaginous overgrowth of the graft surface.

Studies assessing the tribological performance of tissue engineered cartilage constructs have highlighted that the structure and composition of repair tissues play a key role in dictating their frictional response and susceptibility to damage. Although some tissue engineered osteochondral constructs have shown a time-dependent frictional response, they have yet to demonstrate the true biphasic behaviour of native cartilage. There is a clear interdependency between the biomechanical and biotribological properties of osteochondral grafts and their functional performance in the natural joint environment. The biomechanical, biotribological and structural properties of osteochondral grafts ultimately determine their ability to withstand wear and the local biotribology within the joint, therefore preventing or promoting further degenerative changes in the surrounding and opposing articular surfaces.

At present, there is a distinct lack of mechanical and tribological assessment of potential osteochondral repair solutions in order to evaluate their functional performance and efficacy in the natural knee joint. In order to efficiently develop successful osteochondral repair solutions, in vitro and in vivo evaluations should not be purely limited to the assessment of gross morphology, structure and composition. Preclinical evaluations should also assess the mechanical and tribological performance, as functionality is key to producing osteochondral constructs capable of withstanding the complex loading environment of the knee joint while supporting the growth of repair tissue.

In addition to the standard indentation and compression tests generally used to assess material properties, it would be useful to assess the stability of osteochondral repair solutions in the knee joint using push in and push out tests. These methods allow for an assessment of stability and resistance to motion, and they have previously been utilised in published research studies but have been limited to the testing of osteochondral autografts and allografts. Biotribological evaluation of osteochondral repair solutions can provide a better understanding of functional performance than uniaxial biomechanical testing alone. Small-scale biotribological pin-on-plate tests can provide key information regarding the ability of osteochondral repair solutions to restore a biphasic, low friction articulation with negligible wear. Robust preclinical assessment of osteochondral repair solutions may be achieved through the use of whole joint simulators capable of reproducing the natural physiological and anatomical conditions within the knee joint. Whole joint simulation models should allow for the biotribological assessment of repair solutions under dynamic loading profiles and the evaluation of the resulting friction, lubrication and wear in the wider joint. Future studies evaluating performance should include appropriate control groups and comparisons to existing osteochondral repair therapies; where appropriate, these may include experimental groups such as cartilage defects, osteochondral allografts or autografts, disease models and commercially available osteochondral scaffolds.

Robust evaluation of osteochondral repair solutions through both in vitro and in vivo preclinical testing will aid the efficient development of current and future osteochondral repair solutions. A more systematic approach to the assessment of osteochondral repair solutions will allow for easier comparison of functional performance between different regenerative osteochondral repair strategies and to current repair strategies used in the clinic.

## Conclusion

The predicted future population trends indicate a clear requirement for the development of effective therapies for the repair or regeneration of osteochondral defects in the knee. A wide variety of strategies to produce potential regenerative osteochondral repair solutions is currently been researched; however, to date, there has been limited evaluation of the biomechanical and biotribological properties of potential osteochondral repair solutions and their effects within the natural joint environment. The structure and composition of osteochondral repair solutions have been shown experimentally to have a direct impact on the functional performance; therefore, therapies which more closely mimic the structure and composition of natural cartilage and bone tissue are likely to have improved functional properties. In addition to these improvements, the development of an effective, functional osteochondral repair solution for successful delivery to the clinic requires the implementation of robust in vitro preclinical evaluation strategies simulating the in vivo physiological conditions of the natural joint.
